# Inflammatory Manifestations of Lymphedema

**DOI:** 10.3390/ijms18010171

**Published:** 2017-01-17

**Authors:** Catherine L. Ly, Raghu P. Kataru, Babak J. Mehrara

**Affiliations:** The Department of Surgery, Division of Plastic and Reconstructive Surgery, Memorial Sloan Kettering Cancer Center, New York, NY 10065, USA; lyc@mskcc.org (C.L.L.); katarur@mskcc.org (R.P.K.)

**Keywords:** lymphedema, inflammatory skin conditions, inflammatory cells, immunity

## Abstract

Lymphedema results from lymphatic insufficiency leading to a progressive inflammatory process that ultimately manifests as discomfort, recurrent infections, and, at times, secondary malignancy. Collectively, these morbidities contribute to an overall poor quality of life. Although there have been recent advances in microsurgical interventions, a conservative palliative approach remains the mainstay of treatment for this disabling disease. The absence of a cure is due to an incomplete understanding of the pathophysiological changes that result in lymphedema. A histological hallmark of lymphedema is inflammatory cell infiltration and recent studies with animal models and clinical biopsy specimens have suggested that this response plays a key role in the pathology of the disease. The purpose of this report is to provide an overview of the ongoing research in and the current understanding of the inflammatory manifestations of lymphedema.

## 1. Introduction

Lymphedema is a progressive disease resulting from congenital abnormalities, obstruction, injury, or infection of the lymphatic system. Patients with lymphedema have swelling and fibrosis of the affected region resulting in functional problems, decreased quality of life, and recurrent infections [1]. In Western countries, secondary lymphedema is significantly more common and is most often due to lymphatic injury during the course of cancer treatment with risk factors such as extensive lymph node dissection and adjuvant chemoradiation therapy [2]. These complications occur in various solid malignancies, including breast cancer, gynecological tumors, melanoma, and sarcoma [3]. Because the incidence of lymphedema is directly correlated with survival time after oncologic therapy, it is likely that the number of patients who suffer from this disease will increase over time as life expectancy in cancer survivors is improved with better treatments [4]. This is important since it is currently estimated that 5 million Americans are affected [4]. Therefore, lymphedema is a great biomedical burden and research aiming to identify improved treatment options should be a significant goal.

The primary treatment for lymphedema is a conservative approach known as complete decongestive therapy (CDT), which includes physical therapy, manual lymphatic drainage, and skin care [5,6]. These treatments aim to decrease lymphatic fluid accumulation in the tissues by a variety of measures and are palliative in nature, aiming to prevent disease progression rather than cure the underlying pathology. Recent advances exploring pharmacotherapy and surgical treatments have shown some promise [7,8,9]. However, the ideal treatment for this disabling disease has yet to be identified and likely includes a multimodal approach. In order to develop such a treatment, it is necessary to understand the pathophysiological changes that occur following lymphatic injury. Olszewski noted that the pathophysiology involves alterations in tissue morphology, ground substance, lymph biochemistry, lymph immune cells, tissue fluid and lymph pressure and flow, and bacteriology of tissues, but further research is needed to understand the mechanisms by which these changes come about [10].

Studying the mechanisms underlying lymphedema has been complicated by several factors. First, lymphedema develops in a delayed fashion, often years or even decades, after the inciting lymphatic injury [11,12]. Second, not all patients who experience an insult to the lymphatic system go on to develop the disease. Although several risk factors such as radiation, infection, extensive surgical dissection, and obesity have been identified, it remains difficult to predict which patients will display the lymphedema phenotype and how severe their manifestations may be [13,14,15]. These findings suggest that lymphatic injury alone is not sufficient for the development of lymphedema. Rather, it is likely that there are intervening secondary events that contribute to and perpetuate the disease.

Both experimental and clinical studies spanning several decades have implicated inflammation as a critical component in the pathophysiology of lymphedema. Nearly forty years ago, Casley-Smith and Gaffney used rats to conclude that lymphedema results in a chronic inflammatory reaction [16]. More recently, a prospective study of genetic variations of patients diagnosed with breast cancer by Fu et al. found that inflammatory genes were associated with a greater number of symptoms due to lymphedema [13]. With this knowledge, several active laboratories have therefore sought to understand how inflammatory reactions regulate the pathology of this disease. In order to do so, a variety of animal models have been developed, as the need for repeated invasive biopsies and the numerous issues related to the collection, handling, and storage of human tissues precludes the ability to carry out effective time-course studies and histologic analyses using clinical specimens exclusively [17,18,19]. Over the years, animal studies, especially those utilizing mice, have provided significant insight into the cellular and molecular mechanisms of inflammation, much of which has been supported by the characterization of changes in human lymphedematous tissues. This article seeks to provide an overview of the current understanding of the pathophysiology of the inflammatory manifestations of lymphedema.

## 2. Mouse Models

**Figure 1:**
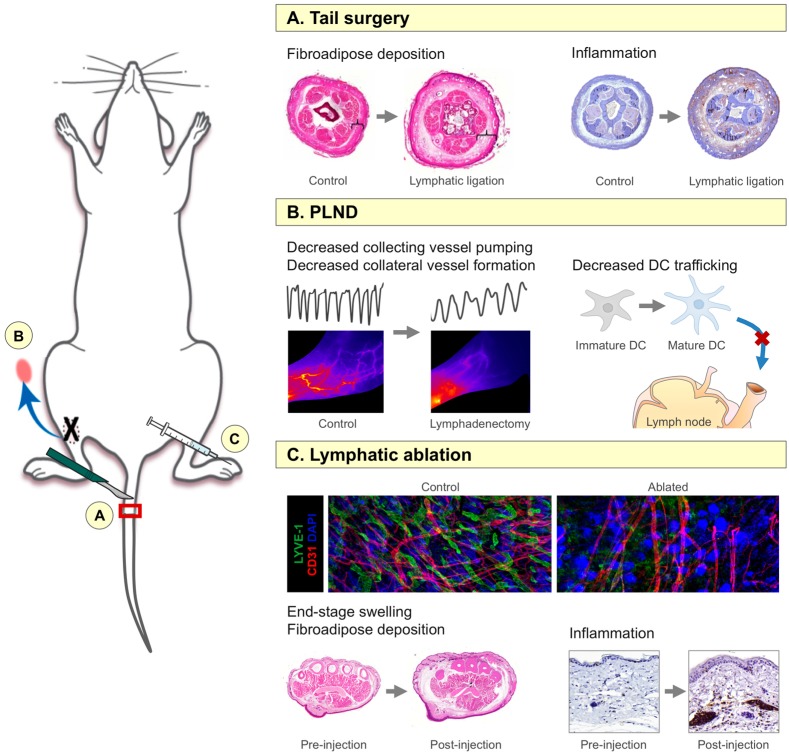
Mouse models of lymphedema. (**A**) Tail surgery model, in which the lymphatics are ligated after circumferential full-thickness skin excision and identification with blue dye. Note the increase in fibroadipose thickness and inflammation following lymphatic injury; inflammatory cells are indicated by the brown color; (**B**) Popliteal lymph node dissection (PLND) model, similar to the axillary lymph node dissection (ALND) model, in which the appropriate lymph node is removed with its corresponding fat pad (as indicated by the blue arrow). This model is particularly useful for evaluation of collecting vessel pumping, collateral lymphatic formation, and dendritic cell (DC) trafficking, all of which are decreased following lymphadenectomy. In the left panel, note the decreased frequency of collecting vessel pumping as indicated by the black line graphs (each peak represents one pump; arbitrary units) and the paucity of patent collecting lymphatic vessels as indicated by absence of distinct vessels highlighted by the red/orange dye in mice that had undergone lymphadenectomy compared to control mice. Lymphatic injury also prevents transport of mature DCs to lymph nodes, where they would initiate immune responses; (**C**) Diphtheria toxin-mediated lymphatic ablation model, in which human diphtheria toxin receptor is coupled with lymphatic-specific receptor promoter Fms-related tyrosine kinase 4 (FLT4) using Cre-Lox technology. After activation with tamoxifen, diphtheria toxin (DT) can be injected into any limb for local ablation. Note the paucity of LYVE-1^+^ lymphatic vessels (green) with preservation of CD31^+^ blood vessels (red); DAPI staining of nuclei is represented in blue. This model also results in prolonged hindlimb swelling and increased inflammation up to one year post-injection; inflammatory cells are indicated by the brown color.

Much of the recent knowledge in lymphedema is built upon the early work of Olszewski, who utilized a canine lymphedema model in his experimental studies [20]. Since this time, many additional animal models, ranging from rodents to larger animals such as sheep and pigs, have been developed in an attempt to more closely simulate human disease [19]. A perfect model does not exist, but mice have been especially valuable for the understanding of lymphedema pathophysiology due to their low cost and the availability of molecular reagents [18]. Three mouse models in particular have been useful in the study of the inflammatory responses incited by lymphatic injury and are therefore described in further detail to provide a background for some of the important studies that have contributed to the current body of literature (Figure 1) [18,19].

The well-described mouse tail surgery model is possibly the most widely utilized means by which to study the cellular and molecular mechanisms of lymphedema [21]. In this model, the superficial and deep lymphatic systems of the tail are microsurgically ligated after circumferentially excising a 3–5 mm portion of the skin and identifying the lymphatic channels by injecting a blue dye (Figure 1A). This results in lymphatic fluid stasis, lymphatic vessel dilation, tail swelling, inflammation, adipose deposition, and fibrosis that closely simulate the histologic features of post-surgical lymphedema in humans (rather than primary or infection-related lymphedema) for at least ten weeks post-operatively [22,23,24,25]. Due to the ease of the procedure, its reproducibility, and its effectiveness, the tail surgery model has become increasingly popular [26]. However, it is limited by the fact that it relies on a relatively large excisional wound, as well as the fact that the tail swelling and fibrosis resolve spontaneously over prolonged periods of time (14–16 weeks). In addition, because no lymph nodes are removed as in the clinical disease, some authors have questioned the clinical relevance of this model in identifying novel therapies for lymphedema [19,27].

In contrast to the tail surgery model, the mouse models of axillary and popliteal lymph node dissection (ALND and PLND, respectively) more closely mimic the clinical scenario of lymph node dissection during oncologic surgery. In these models, a popliteal or axillary skin incision is made and respective lymph nodes are dissected out and removed with their corresponding fat pads (Figure 1B). Using near-infrared imaging, Blum et al. found that removal of the lymph node with its corresponding fat pad (rather than just the lymph node alone) results in collecting vessel rupture, dermal backflow, and lymphatic fluid drainage through collateral vessels, similar to that noted following ALND during breast cancer surgery [28]. More recent studies have suggested that, compared to the tail surgery model, these lymphadenectomy models are better suited for the analysis of the regeneration of collateral lymphatics, collecting lymphatic pumping capacity, and dendritic cell (DC) trafficking [22,29]. They also enable histologic evaluation of the lymph nodes themselves and their interactions with inflammatory cytokines and antigen-presenting cells (APCs), in addition to permitting testing of preventative therapies for lymphedema. However, similar to the tail surgery model, sustained edema is often not observed and there is little fibrosis or adipose deposition in the subcutaneous tissues (similar to the acute effects of lymph node dissection clinically), therefore limiting the utility of this model in testing novel therapies for chronic lymphedema.

In order to avoid the pitfalls of previous animal models, our laboratory recently reported on a new mouse model in which lymphatic endothelial cells (LECs) are non-surgically ablated with resultant progressive lymphedema (Figure 1C) [30]. Using Cre-Lox technology, human diphtheria toxin receptor (DTR) is coupled with a lymphatic-specific promoter known as Fms-related tyrosine kinase 4 (FLT4), thereby allowing for ablation of LECs of the entire lymphatic tree through DTR activation. Gardenier et al. found that this model closely simulates clinical findings in which acute post-surgical edema spontaneously resolves over several weeks with subsequent development of chronic and progressive lymphedema for at least one year [30]. In addition to matching the temporal sequence of clinical lymphedema, the Cre-Lox model also mimics the human disease histologically and radiographically, as the initial resolution of the edema correlates with the infiltration of M2-polarized macrophages, whereas the subsequent accumulation of CD4^+^ cells results in impaired lymphangiogenesis, collecting fibrosis, and lymphatic smooth muscle cell proliferation associated with lymphedema. The importance of these inflammatory changes will be discussed in greater detail in the following sections. By utilizing these transgenic mice in conjunction with the tail surgery and lymphadenectomy models, it is possible to more accurately study the complex interactions that initiate and propagate the pathology of lymphedema and develop therapies that are effective in both prevention and treatment of this disease.

## 3. Inflammatory Manifestations of Lymphedema

Fluid flow through the lymphatic vasculature plays a critical role in fluid homeostasis, immunity, and lipid reabsorption [31,32]. When lymphatic injury is present, fluid flow is disrupted, leading to fluid accumulation, vessel distention, valve dysfunction, and reflux [33,34]. Changes in normal tissue function through inflammation, tissue remodeling, lymphatic hyperplasia, and adipocyte deposition subsequently occur, thus resulting in the lymphedema phenotype [35]. As suggested by Rutkowski and Swartz, the increased fluid flow itself may serve as an early signaling cue of inflammation that triggers surrounding fibroblasts to initiate rapid matrix repair through autocrine upregulation of transforming growth factor-β1 (TGF-β1), differentiation into myofibroblasts, and increased collagen production and alignment [35]. If collateral lymphatics are unable to compensate for the initial lymphatic injury, the resultant persistence of fluid accumulation in the interstitial space contributes to an ongoing positive feedback loop of inflammation that ultimately leads to the pathologic changes of lymphedema. Continued remodeling of the extracellular matrix alters soft-tissue compliance and decreases lymphatic function, eventually leading to the obliteration of lymphatic vessels, as seen in advanced stages of the disease [23].

### 3.1. Upregulation of Inflammatory Genes and Proteins

Gene expression analysis and protein quantification studies have shown that the expression of pro-inflammatory genes is upregulated in animal models and patients with lymphedema [36,37,38,39]. In a study of affected patients undergoing CDT, for example, Foldi et al. noted that that the expression of pro-inflammatory genes such as that for CD14, interferon-gamma (IFN-γ) receptor, tumor necrosis factor-alpha (TNF-α), integrin alpha 4 beta 1 (α4β1; also known as Very Late Antigen-4 or VLA-4), tumor necrosis factor receptor p55 (TNFR1), and CD44 were increased prior to and significantly decreased after the first phase of treatment [36]. Similarly, Tabibiazar et al. utilized transcriptional profiling of lymphedematous tissues from a mouse tail surgery model to find that the upregulated genes were similar to those noted in acute inflammation and wound healing [25]. The same group also found that the gene expression pathways invoked by human lymphedema included those involved in T receptor signaling, cytokine expression, and antigen processing and presentation [37].

In addition, Zampell et al. described increased expression of endogenous danger signals high-mobility group box 1 (HMGB1) and heat shock protein 70 (HSP70) in tissues from both the ALND mouse model and matched human lymphedema biopsy samples and hypothesized that these early responses promote chronic inflammatory changes [29]. The same investigators also determined that deficiency of the toll-like receptors activated by these endogenous danger signals worsens inflammatory tissue responses following lymphatic injury [40].

### 3.2. CD4^+^ Cell Inflammation

Several groups have attempted to characterize the inflammatory response in lymphedema [21,22,41,42,43,44,45,46]. Recent studies from at least three laboratories have demonstrated a role for CD4^+^ cells in the pathophysiology [22,43,44,47]. In two separate studies using the mouse tail surgery model and lymph node dissection models, respectively, Avraham et al. and Zampell et al. showed that lymphatic injury results in a mixed inflammatory response, of which greater than 70% are CD4^+^ cells [22,41]. Avraham et al. also reported similar changes in biopsy specimens collected from patients with unilateral upper extremity breast cancer-related lymphedema. More importantly, they found that the number of tissue-infiltrating CD4^+^ cells had a positive linear correlation with the severity of disease. Furthermore, using the tail model of lymphedema in nude mice (which lack all T cells) and CD4 knockout mice (which lack CD4^+^ cells), Avraham et al. and Zampell et al. found that the absence of T cells in general and CD4^+^ cells in particular was protective for the development of lymphedema. Knockout mice had significantly decreased swelling, subcutaneous tissue thickness, CD45^+^ infiltration, and fibrosis, as well as improved lymphatic function. These authors reported similar changes when CD4^+^ cells were depleted using neutralizing antibodies in wild-type mice. This effect was specific for CD4^+^ cells since depletion of CD8^+^ or CD25^+^ cells using similar methods did not prevent development of lymphedema [41]. In a recent study from another group that also utilized the mouse tail surgery model, Gousopoulos et al., reported that the lymphatic vessel remodeling and collecting vessel impairment in lymphedema correlated with increased numbers of immune cells, particularly Ly6G^+^ and CD4^+^ cells [47]. Similarly, Ogata et al. found that CD4^+^ T cells interact with macrophages, leading to vascular endothelial growth factor C (VEGF-C) expression, which then promotes the generation of immature lymphatic vessels that are essential for the development of initial edema [43]. Taken together, the collective findings of these studies from three separate laboratories suggest that lymphedema results in increased tissue infiltration of CD4^+^ cells in lymphedematous tissues of mice and humans. In addition, neutralizing studies and experiments with knockout animals suggest that these cells play a key role in the pathophysiology of lymphedema.

### 3.3. T Helper Cells

Fibrosis is a histological hallmark of lymphedema, as patients with the disease present with fibrosis of the skin and subcutaneous tissues associated with increased collagen deposition [1]. In addition, recent studies have shown that lymphatic collecting vessels in the affected limb become progressively fibrosed and replaced by scar tissue that obliterates the lumen area of the vessel [48]. These features suggest that lymphedema may represent fibrotic organ failure of the lymphatic system. In fact, fibrosis is a common mode of end-organ failure and can affect virtually any major organ system, including liver, lung, heart, pancreas, and kidney. A large body of literature has shown that the cellular mechanisms that regulate fibrosis in a variety of organ systems is preserved and, in large part, regulated by T helper cells [49,50]. These studies have led to the description of the T helper 1 (Th1) and T helper 2 (Th2) paradigm, postulating that pro-fibrotic cytokines and growth factors elaborated by Th2 cells, including interleukin 4 (IL-4), IL-13, and TGF-β1, regulate collagen deposition and fibrosis in widely disparate pathologies [49,50].

**Figure 2:**
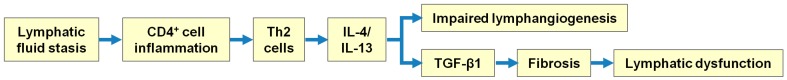
Pathophysiologic sequence following CD4^+^ cell inflammation in lymphedema. A compromised lymphatic system leads to lymphatic fluid stasis, which subsequently contributes to the accumulation of a distinct CD4^+^ inflammatory infiltrate. Although there is a mixed Th1 and Th2 response, Th2 cells in particular have been found to be critical in the development of the pathologic findings of lymphedema. Th2 cytokines IL-4 and IL-13 contribute to lymphedema through the impairment of lymphangiogenesis and upregulation of TGF-β1, which promotes fibrosis. The accumulation of fibrotic components over time ultimately leads to lymphatic dysfunction.

Evidence derived from experimental and clinical specimens suggest that Th2 cells also play a key role in the regulation of fibrosis and lymphatic dysfunction in lymphedema [22,41]. Using a variety of techniques, including immunofluorescent staining and flow cytometry using tissues from the mouse tail surgery and ALND models, as well as clinical biopsy specimens collected from patients with unilateral upper extremity breast cancer-related lymphedema, Avraham et al. and Zampell et al. found that lymphatic injury resulted in a Th2-biased response with infiltration of large numbers of CD4^+^ IL-4^+^ IL-13^+^ Th2 cells (Figure 2) [41,51]. Because Th2 differentiation is dependent on IL-4 or IL-13 signaling, Avraham et al. utilized monoclonal IL-4 and IL-13 antibodies in the mouse tail surgery model and found that blockade of these cytokines prevented development of lymphedema after tail injury and resulted in significant improvement in animals with established disease [41]. Treated animals had markedly decreased swelling, fibrosis, and adipose deposition, as well as significant improvements in lymphatic transport function. In contrast, non-specific anti-inflammatory treatments or blockade of different inflammatory pathways (i.e., STAT3) had no effect on lymphedema development.

Other studies using the mouse tail surgery and lymphatic ablation models and clinical biopsy specimens have suggested that TGF-β1 also plays a key role in the regulation of fibrosis and lymphatic dysfunction in lymphedema [23,24,30]. These investigators showed that TGF-β1 and its downstream mediator phosphorylated SMAD 3 (pSMAD3) are increased in lymphedematous tissues in both mice and patients. Inhibition of TGF-β1 signaling using monoclonal neutralizing antibodies or transmission of dominant negative TGF-β1 receptors using adenoviral vectors markedly decreased fibrosis and improved lymphatic function in mouse models [23,24]. Consistent with fibrosis in other organ systems, the studies also suggest that TGF-β1 signaling has a reciprocal interaction with Th2 cells, as inhibition of TGF-β1 also markedly decreased T cell infiltration and Th2 differentiation [23,24,49,50].

Th1 and Th2 cytokines, as well as TGF-β1 signaling, may also regulate lymphatic function via other mechanisms. For example, Savetsky et al. found that, in addition to having profound pro-fibrotic activity, IL-4 and IL-13 also impair LEC survival, proliferation, migration, and tubule migration in both in vitro and in vivo studies [51]. Similarly, Shin et al. found that these Th2 cytokines downregulate LEC-specific transcription factor Prox-1 and LEC marker LYVE-1 and that blockade of these cytokines improves both lymphatic vessel formation and function both in vitro and in an asthma model [52]. The authors also demonstrated that the anti-lymphangiogenic effects of IL-4 and IL-13 are predominant even in environments rich in pro-lymphangiogenic factors. Of note, IFN-γ, a key Th1 cytokine, also has profound anti-lymphangiogenic activity in vitro and in vivo, with studies demonstrating its negative effects on lymphatic sprouting and inflammatory lymphangiogenesis [46,53]. In addition, other researchers have shown that TGF-β1 has potent anti-lymphangiogenic activity in a variety of settings with direct inhibition of LEC differentiation, migration, and tubule formation, even when cells are cultured with high concentrations of VEGF-C [24,54]. Taken together, these findings suggest that fibrosis as mediated by Th2 cytokines is critical to the pathology of lymphedema, likely due to the progressive obliteration of the superficial and deep lymphatic systems with worsening lymphatic function and inadequate collateral lymphatic growth [48,55].

### 3.4. T Regulatory Cells

Studies of the CD4^+^ cells in lymphedema have shown that T regulatory cells (Tregs) comprise a notable proportion of the inflammatory infiltrate in both mouse models of lymphedema and human biopsy samples [22]. RNA sequencing of lymphadematous mouse tissues have also revealed upregulation of the transcription factor Foxp3 [56]. In an effort to elucidate the role of these cells, Zampell et al. demonstrated that treating mice with neutralizing antibodies to deplete Tregs did not result in an improvement in the lymphedema phenotype [22]. However, Gousopoulos et al. found that the absence of Tregs correlated with exacerbated edema and increased infiltration of immune cells instead [56]. Furthermore, the authors utilized adoptive Treg transfer following mouse tail surgery with resultant improvement in swelling, inflammation, fibrosis, and lymphatic remodeling and function. Similarly, in a study of lymphatic filariasis, Wammes et al. noted that in vitro depletion of Tregs led to increased Th2 cytokine responses [57]. Such findings are supported by previous studies demonstrating the critical role that Tregs play in homeostasis through negative regulation of immune-mediated inflammation [58].

Although Tregs serve an important purpose by attenuating the severity of inflammatory tissue responses in lymphedema, it is likely that these cells also contribute to the local impaired adaptive immune response manifested by the occurrence of recurrent soft tissue infections in lymphedema [56,59,60]. Further studies will need to be done to elucidate the mechanisms by which this immune dysregulation occurs.

### 3.5. Macrophages

Given the known role of macrophages in inflammation and fibrosis, it is not surprising that studies have also noted significant macrophage accumulation in lymphedema [22,29,42,61]. Rutkowski et al., for example, noted that macrophages were present throughout the dermis and hypodermis in edematous skin for at least four weeks following mouse tail surgery [21]. Studies have also shown that macrophages produce and activate TGF-β1 [61]. The role of macrophages in regulating lymphedema pathology is complex, however, as Ghanta et al. have demonstrated that macrophage depletion in the tail surgery model significantly increases fibrosis, suggesting that macrophages serve a counteracting anti-fibrotic role as well [40,42]. Furthermore, macrophages were noted to either directly or indirectly regulate CD4^+^ accumulation and subsequent Th2 differentiation, suggesting that there is an interplay between the inflammatory cell types [41,51,52]. These findings are consistent with studies showing that macrophages become alternatively activated as M2 macrophages in response to IL-4 and IL-13, that M2 macrophages can be generalized as immunosuppressive, and that macrophages can serve opposing purposes depending on environment, timing, and location [42,62,63].

Other studies have highlighted the role of macrophages in the regulation of inflammatory lymphangiogenesis by elaborating lymphangiogenic growth factors including VEGF-A and VEGF-C in a variety of settings [43,45,64,65,66]. Some researchers also have suggested that macrophages are capable of transdifferentiating into LECs during inflammatory lymphangiogenesis [67,68]. More recently, Ghanta et al. provided evidence that macrophage depletion, after the establishment of lymphedema, leads to decreased lymphatic transport activity and VEGF-C expression [40,42]. In addition, using the depletion studies with the mouse lymphatic ablation model, Gardenier et al. found that M2 macrophages are likely key regulators of the regeneration of collateral lymphatics after lymphatic injury [30].

Macrophages are also a significant source of IL-6, a cytokine that has been shown to play an important role in the regulation of chronic inflammation and adipose metabolism [69]. Using loss-of-function experiments, Cuzzone et al. demonstrated that upregulation of IL-6 in mouse models of lymphedema acts to decrease adipose deposition [70]. Similarly, Karlsen et al. found that genetically engineered Chy mice, a model for primary congenital lymphedema, had elevated levels of IL-6 at later stages of the disease [71]. These findings are supported by clinical studies showing elevated levels of IL-6 in the lymphedematous tissues of patients [10,39,70].

Furthermore, macrophages are also known to promote the expression of inducible nitric oxide synthase (iNOS), which attenuates lymphatic vessel contraction in inflammation [72,73,74,75]. Liao et al. showed that iNOS causes a reduction in the strength of lymphatic contraction through excessive relaxation of collecting lymphatic vessels and that inhibition of iNOS-producing cells significantly restores lymphatic contraction [73]. The resultant impairment in lymphatic transport not only leads to the accumulation of lymphatic fluid, but also contributes to immunosuppression by reducing antigen transport to lymph nodes [75]. Taken together, these studies suggest that macrophages play a complex role in the pathophysiology of lymphedema by directly and indirectly regulating fibroadipose tissue deposition, lymphangiogenesis, and lymphatic vessel pumping.

### 3.6. Dendritic Cells

DCs comprise a major proportion of lymph fluid and are responsible for the presentation of peripheral antigens to immune effector cells in regional lymph nodes [76]. Following induction of migration by pro-inflammatory cytokines such as IL-1 and TNF-α, DCs produce digestive enzymes such as metalloproteinases to aid their passage through the basement membrane and extracellular matrix in order to reach lymphatic vessels [76]. However, in the setting of lymphatic injury in which afferent lymphatic vessels are either severed or destroyed, DCs that have matured in the periphery are unable to migrate normally. For example, using a mouse tail surgery model, Rutkowski et al. found that Langerhans DCs had delayed migration through the dermis of edematous skin, possibly due to the absence of migration-directing flow [21]. This subsequently leads to a persistence of DCs in the peripheral tissue, where they can produce pro-inflammatory mediators that contribute to the ongoing cycle of inflammation [77,78].

### 3.7. Homing of Inflammatory Cells to Lymphedematous Tissues

The molecular pathways leading to the accumulation of the distinct inflammatory infiltrate in lymphedema are not yet fully elucidated. Our ongoing work suggests that lymphatic injury through lymph node dissection leads to activation and release of CD4^+^ cells from regional lymph nodes and that these cells have increased expression of skin-homing receptors cutaneous lymphocyte antigen (CLA), C–C motif chemokine receptor 4 (CCR4), and CCR10 (unpublished). In addition, the leukocyte adhesion molecules endothelial cell selectin (E-selectin) and platelet selectin (P-selectin), which are ligands for CLA, as well as the chemokines CCL17 and CCL27, which are the ligands for CCR4 and CCR10 respectively, have also been found to be notably increased in lymphedematous skin (unpublished). Additional studies will be necessary to further delineate the importance of these and other molecules promoting the accumulation of CD4^+^ cells.

### 3.8. Application of Knowledge from Mouse Models to Human Lymphedema

The delayed presentation and natural history of lymphedema make it difficult to study the pathophysiology of this disease without animal models. Transcriptional profiling and histologic analysis of clinical specimens have shed light on specific pathways and the immune cells that participate in them, but time-course studies using readily available and reliably reproducible animal models have been critical in mechanistic studies. Importantly, parallel analysis of clinical biopsy samples by multiple laboratories has shown evidence of increased populations of CD4^+^ cells such as Th2 cells and Tregs in human lymphadematous tissues, thus putting the findings from the mouse models into context and allowing for appropriate clinical translation [22,41,56].

## 4. Conclusions

Secondary lymphedema results when the complex series of molecular interactions with intricately designed regulatory feedback mechanisms following lymphatic injury tips the balance against effective lymphangiogenesis and in favor of chronic inflammation, fibrosis, and immunosuppression. The absence of lymphedema in some patients despite lymphatic injury and the delayed development of the disease in others indicates that secondary events are likely necessary to elicit these important pathologic interactions [12]. Current evidence suggests that a variety of key players, including T helper cells, Tregs, macrophages, and dendritic cells, play complex roles in the pathology of the disease by elaborating inflammatory cytokines and regulating the development of collateral lymphatic vessels (Table 1). Further studies will need to be conducted to more clearly understand the interactions between these cells and how inflammatory changes regulate the development of lymphedema.

## Figures and Tables

**Table 1:** Summary of selected cytokines and growth factors involved in lymphedema.

Cytokine	Function
IFN-γ	Impairs lymphangiogenesis
	Activates macrophages
IL-1	Induces DC migration
IL-13	Impairs lymphangiogenesis
	Promotes M2 macrophage activation
IL-4	Impairs lymphangiogenesis
	Promotes M2 macrophage activation
IL-6	Regulates chronic inflammation
	Decreases adipose deposition
TGF-β1	Mediates soft-tissue fibrosis
	Negatively regulates lymphatic vessel regeneration
VEGF-C	Promotes lymphangiogenesis

## Abbreviations

CDT	complete decongestive therapy
ALND	axillary lymph node dissection
PLND	popliteal lymph node dissection
DC	dendritic cell
APC	antigen-presenting cell
LEC	lymphatic endothelial cell
DTR	diphtheria toxin receptor
FLT4	Fms-related tyrosine kinase 4
TGF-β1	transforming growth factor-β1
IFN-γ	interferon-gamma
TNF-α	tumor necrosis factor-α
α4β1	alpha 1 beta 4
VLA-4 TNFR1	very late antigen-4 tumor necrosis factor receptor p55
HMGB1	high-mobility group box 1
HSP70	heat shock protein 70
VEGF-C	vascular endothelial growth factor C
Th1	T helper 1
Th2	T helper 2
IL-4	interleukin-4
IL-13	interleukin-13
pSMAD3	phosphorylated SMAD3
Tregs	T regulatory cells
VEGF-A	vascular endothelial growth factor A
iNOS	inducible nitric oxide synthase
IL-1	interleukin-1
CLA	cutaneous lymphocyte antigen
CCR4	C–C motif chemokine receptor 4
CCR10	C–C motif chemokine receptor 10
E-selectin	endothelial cell selectin
P-selectin	platelet selectin
CCL17	C–C motif chemokine ligand 17
CCL27	C–C motif chemokine ligand 27
